# Vanishing twin syndrome among ART singletons and pregnancy outcomes

**DOI:** 10.1093/humrep/dex277

**Published:** 2017-08-31

**Authors:** Maria C Magnus, Sara Ghaderi, Nils-Halvdan Morken, Per Magnus, Liv Bente Romundstad, Rolv Skjærven, Allen J Wilcox, Siri Eldevik Håberg

**Affiliations:** 1 Division for Mental and Physical Health, Norwegian Institute of Public Health, P.O. Box 4404 Nydalen, N-0403 Oslo, Norway; 2 MRC Integrative Epidemiology Unit at the University of Bristol, Oakfield House, Oakfield Grove, BS8 2BN Bristol, UK; 3 School of Social and Community Medicine, University of Bristol, Oakfield House, Oakfield Grove, BS8 2BN Bristol, UK; 4 Department of Global Public Health and Primary Care, University of Bergen, P.O. Box 7804, N-5020 Bergen, Norway; 5 Department of Clinical Science, University of Bergen, P.O. Box 7804, N-5020 Bergen, Norway; 6 Centre for Fertility and Health (CeFH), Centre of Excellence at Norwegian Institute of Public Health, P.O. Box 4404 Nydalen, N-0403 Oslo, Norway; 7 Institute for Health and Society, University of Oslo, P.O. Box 1130 Blindern, N-0403 Oslo, Norway; 8 Department of Obstetrics and Gynaecology, IVF Unit, St Olav's University Hospital, P.O. Box 3250 Sluppen, N- 7006 Trondheim, Norway; 9 Department of Public Health and Nursing, Norwegian University of Science and Technology, P.O. Box 8905, N-7491 Trondheim, Norway; 10 Division for Health Data and Digitalization, Norwegian Institute of Public Health, P.O. Box 4404 Nydalen, N-0403 Oslo, Norway; 11 Epidemiology Branch, National Institute of Environmental Health Sciences, National Institutes of Health, Research Triangle Park, North Carolina NC 27709, USA

**Keywords:** vanishing twin syndrome, birth weight, gestational age, Centre for Fertility and Health, Norway, ART, small for gestational age

## Abstract

**STUDY QUESTION:**

Among babies born by ART, do singleton survivors of a vanishing twin have lower birth weight than other singletons?

**SUMMARY ANSWER:**

Vanishing twin syndrome (VTS) was associated with lower birth weight among ART singletons; a sibship analysis indicated that the association was not confounded by maternal characteristics that remain stable between deliveries.

**WHAT IS KNOWN ALREADY:**

Previous studies indicate that ART singletons with VTS have increased risk of adverse pregnancy outcomes, compared with other ART singletons. The potential contribution of unmeasured maternal background characteristics has been unclear.

**STUDY DESIGN, SIZE AND DURATION:**

This was a Norwegian population-based registry study, including 17 368 mothers with 20 410 ART singleton deliveries between January 1984 and December 2013.

**PARTICIPANTS/MATERIALS, SETTING, METHODS:**

The study population included 17 291 ART singletons without VTS, 638 ART singletons with VTS and 2418 ART singletons with uncertain vanishing twin status. We estimated differences in birth weight and gestational age comparing ART singletons with VTS first to all ART singletons without VTS, and subsequently to their ART siblings without VTS, using random- and fixed-effects linear regression, respectively. The corresponding comparisons for the associations with preterm birth and small for gestational age (SGA) were conducted using random-and fixed-effects logistic regression. The sibling analysis of preterm birth included 587 discordant siblings, while the sibling analysis of SGA included 674 discordant siblings.

**MAIN RESULTS AND THE ROLE OF CHANCE:**

ART singletons with VTS had lower birth weight when compared to all ART singletons without VTS, with an adjusted mean difference (95% CI) of −116 g (−165, −67). When we compared ART singletons with VTS to their ART singletons sibling without VTS, the adjusted mean difference was −112 g (−209, −15). ART singletons with VTS also had increased risk of being born SGA, with an adjusted odds ratio (OR) (95% CI) of 1.48 (1.07, 2.03) compared to all ART singletons without VTS, and 2.79 (1.12, 6.91) in the sibship analyses. ART singletons with VTS were also more likely to be born preterm, although this difference did not reach statistical significance.

**LIMITATIONS REASONS FOR CAUTION:**

We did not have information on maternal socio-economic status, but this factor is accounted for in the sibship analyses. We also had no information on whether fresh or frozen embryos were replaced.

**WIDER IMPLICATIONS OF THE FINDINGS:**

The reduction in birth weight and increased risk of SGA in ART singletons with VTS may suggest the presence of harmful intrauterine factors with long-term health impact. While vanishing twins are not routinely observed in naturally conceived pregnancies, loss of a twin is potentially a risk factor for the surviving foetus in any pregnancy. This could be further explored in large samples of naturally conceived pregnancies with the necessary information.

**STUDY FUNDING/COMPETING INTEREST(S):**

The authors of this study are supported in part by the UK Medical Research Council, US National Institute of Environmental Health Sciences and the Norwegian Research Council. The authors have no conflicts of interest.

**TRIAL REGISTRATION NUMBER:**

N/A.

## Introduction

There is an increased use of ART world-wide, including IVF and ICSI, with one in four couples requiring assistance to conceive according to WHO estimates (World Health Organization, 2012). Children born after ART are at increased risk of adverse pregnancy outcomes, including preterm birth, low birth weight, congenital malformations, pregnancy-induced hypertension/pre-eclampsia and delivery by caesarean section (Hansen *et al.*, 2013; Pinborg *et al.*, 2013; Qin *et al.*, 2015, 2016; Bartsch *et al.*, 2016). Similar increased risk of adverse pregnancy outcomes among ART offspring is observed in Norway (Romundstad *et al.*, 2008).

Vanishing twin syndrome (VTS), defined as the spontaneous reduction of a foetus while still in utero (Pinborg *et al.*, 2006), is estimated to occur in 50% of pregnancies that start with three or more gestational sacs, and 36% of twin pregnancies (Dickey *et al.*, 2002). With regard to IVF/ICSI pregnancies, it is estimated to occur in 12–30% (Gjerris *et al.*, 2012). Furthermore, VTS is associated with adverse pregnancy outcomes for the remaining survivor among both spontaneously conceived (Evron *et al.*, 2015) and ART pregnancies (Pinborg *et al.*, 2005, 2007; Shebl *et al.*, 2008; Luke *et al.*, 2009; Almog *et al.*, 2010; Sazonova *et al.*, 2011; Davies *et al.*, 2016; Petrini *et al.*, 2016; Zhou *et al.*, 2016). Adverse outcomes include higher risk of low birth weight, preterm birth, small for gestational age (SGA) and birth defects.

None of these studies could rule out the possibility of confounding by characteristics of the mother. A sibling comparison design (which compares siblings of the same mother) is useful to evaluate the role of unmeasured confounding by maternal background characteristics that remain stable between deliveries (Susser *et al.*, 2010; Frisell *et al.*, 2012). In Norway, all citizens are assigned a unique identification number at birth that enables identification of all pregnancies to a woman in the Medical Birth Registry. This provides a valuable opportunity to conduct comparisons of pregnancy complications and perinatal outcomes within sibships.

The objective of the current study was therefore to estimate the associations of VTS among ART singletons with gestational age and birth weight in the population overall, and also within sibships.

## Materials and Methods

### The Medical Birth Registry of Norway

The Medical Birth Registry of Norway (MBRN) contains information on all births occurring after 16 gestational weeks (Irgens, 2002). Registration of the delivery is mandatory, and is done by a midwife or the attending obstetrician. The registry includes civil data on the mother, father and the offspring, information on maternal health before and during pregnancy, birth outcomes, and pregnancy complications. The MBRN also contains information from all clinics offering ART, including information on the number of fetuses detected on the early ultrasound scan conducted between 6 and 8 gestational weeks. The first ART birth in Norway was recorded in the registry in 1984. We defined our eligible population as offspring of mothers who had their first delivery (excluding spontaneous abortions) between January 1984 and December 2013 (*n* = 1 582 475). The main study population consisted of 17 368 women with 20 410 ART singletons. The number of deliveries per woman ranged from 1 to 4 (mean 1.2), with 2932 women having two or more ART deliveries. Secondary analyses also included information on all ART multiples born in Norway during this period (*n* = 9146).

### Vanihing twin syndrome

We defined ART pregnancies by a recorded procedure of ‘IVF’, ‘ICSI’ or ‘combinations/other’ in the MBRN birth record. VTS was defined among ART pregnancies by comparing the number of fetuses observed on the early ultrasound (5–8 weeks of gestation) with the number of offspring delivered. When information from the early ultrasound was missing, the status of VTS was defined as ‘uncertain’. We compared pregnancy outcomes among ART singletons without VTS (reference), with VTS and with uncertain vanishing twin status.

### Pregnancy outcomes

The outcomes of interest included gestational age, birth weight, preterm birth and SGA. Gestational age at birth (in days) was determined by ultrasound examination from around week 17 of pregnancy, or by date of last menstrual period in absence of ultrasound information (18%). We defined preterm birth as a delivery before 37 completed gestational weeks (<259 days). Birth weight was recorded in grams and kept as a continuous variable in the analyses. We subsequently considered internally standardized birth weight by gestational age, sex and parity, as calculated based on the distribution across all deliveries in Norway within the study period. SGA was similarly defined as birth weight below the 10th percentile using all births in Norway.

### Statistical analysis

For continuous outcomes, we used fixed-, between- and random-effects linear regression models, reporting mean differences and 95% CI. Fixed-effect linear regression provides the within-sibship associations, in which the pregnancy outcomes between ART offspring with and without VTS of the same mother are compared. The within-sibship associations therefore provide estimates of the associations that are unbiased by unmeasured maternal background characteristics that remain stable between deliveries. The between-sibship association compares the pregnancy outcomes among ART offspring with and without VTS between unrelated individuals. This estimate uses data from all participants, but compares the mean pregnancy outcomes within a cluster (mother) to the mean exposure among clusters (mothers). The random-effects linear regression gives the overall association between VTS and pregnancy outcomes comparing all ART singletons, and is a weighted average of the within- and between-sibship associations, where each coefficient is weighted by the inverse of its variance (Mann *et al.*, 2004).

For the binary outcomes, we used fixed- and random-effects logistic regression, reporting odds ratios (ORs) and 95% CI. The coefficients from the fixed-effects logistic regression provide estimates of the within-sibship association, comparing offspring of the same mother, and is restricted to sibships with at least one sibling pair discordant for the outcome of interest. The model is equivalent to conditional logistic regression used for matched case-control studies, where the discordant sibling pairs are the matched pairs of interest (Austin, 2010). The associations from the random-effects logistic regression yields the overall association between VTS and pregnancy outcomes comparing all ART singletons, while also incorporating familial clustering into the estimation of the confidence intervals.

In all adjusted models, we adjusted for maternal age at delivery, parity, marital status, year of birth and chronic diseases during pregnancy (asthma, hypertension, heart disease, kidney disease, rheumatoid arthritis, thyroid disease and/or diabetes). Maternal age at delivery and year of birth were entered as continuous covariates, while the other covariates were categorized as indicated in Table I and entered as dummy variables. All covariates were allowed to vary from pregnancy to pregnancy, as they are unique to the birth record for each delivery in the MBRN. We further adjusted for maternal smoking during pregnancy in a sensitivity analysis restricted to ART offspring born in 1999 or after (when smoking was first registered in the MBRN). We did not have information on maternal socio-economic status. Since this is likely to remain relatively stable between deliveries, this potential source of bias is accounted for in the sibship analyses.

**Table I:** Overview of background characteristics by VTS among ART singleton deliveries born in Norway between July 1984 and December 2013 (*n* = 20 410).

Characteristics	ART singleton without VTS (*n* = 17 291) %	ART singleton with VTS (*n* = 638) %	ART singleton uncertain VTS (*n* = 2481) %
Maternal age (*y*)			
25–29	21	16	21
30–34	43	40	40
35 and older	37	44	39
Maternal parity			
0	65	63	70
1	28	30	25
2 or more	6	7	5
Maternal marital status			
Married/cohabitating	98	98	96
Single	2	2	4
Maternal chronic diseases during pregnancy^a^			
No	88	87	88
Yes	12	13	12
Year of birth			
<1992	2	2	7
1992–1998	10	15	15
1999–2006	31	42	36
2007 and later	57	40	42
Sex			
Male	52	49	49
Female	48	50	50
Uncertain	0.1	0.3	0.2

When information from the early ultrasound was missing, the status of vanishing twin syndrome (VTS) was defined as ‘uncertain’.

^a^Chronic medical conditions included asthma, hypertension, heart disease, kidney disease, rheumatoid arthritis, epilepsy, thyroid disease and diabetes.

The *χ*^2^ tests comparing the distribution of background characteristics between the groups of ART singletons yielded *P*-values < 0.001. The only exception was maternal chronic diseases during pregnancy, which showed no difference across the groups of ART singletons (*χ*^2^*P*-value 0.8).

We conducted a sensitivity analysis in which gestational age was estimated based on date of birth minus the date of the ART procedure (plus 14 days) for the offspring whose date of the ART procedure was available (87%). To further explore the influence of our definition of VTS, we re-examined the associations defining VTS based on the number of foetuses with heart beats detected on the early ultrasound (available for 70%), rather than the number of foetuses observed. As a secondary analysis, we also compared the three groups of ART singletons to ART multiples (reference). All analyses were conducted using Stata version 14 (Statcorp, Texas).

## Results

Table I shows the background characteristics among all ART singletons born in Norway between July 1984 and December 2013—20 410 ART singleton deliveries born to 17 368 mothers. These ART deliveries included 17 291 (85%) singletons without VTS, 638 (3%) singletons with VTS and 2481 (12%) singletons with uncertain vanishing twin status. ART singletons with VTS had older mothers and were more likely to be born before 2007 (Table I). The proportion of ART offspring with VTS did not differ by ART treatment type (4% for IVF offspring and 3% for ICSI offspring).

The mean gestational age for the ART singletons included in the study was 276.6 days (SD 16.7), while the mean birth weight was 3438 g (SD 641). ART singletons with VTS had lower birth weight (both crude and standardized) than those without VTS. This difference persisted when we compared ART offspring with VTS to their ART siblings without VTS (sibship analyses) (Table II). ART singletons with VTS also had shorter gestations but this association was attenuated when evaluated within sibships (Table II).

**Table II:** Birth weight and gestational age among ART singletons with VTS compared to ART singletons without VTS, for all ART singletons born in Norway between July 1984 and December 2013.

Outcome	VTS	*N*	Mean (SD)	Overall analyses^a^		Sibship analyses^b^	
				Unadjusted	Adjusted^c^	Unadjusted	Adjusted^c^
				Mean difference	Mean difference	Mean difference	Mean difference
				(95% CI)	(95% CI)	(95% CI)	(95% CI)
Gestational age in days	No	16 038	276.6 (16.6)	Ref	Ref	Ref	Ref
	Yes	583	275.0 (18.5)	−1.5 (−2.9, −0.2)	−1.4 (−2.7, 0.0)	−0.6 (−3.6, 2.4)	−0.6 (−3.6, 2.4)
	Uncertain^d^	2305	277.2 (16.7)	0.6 (−0.1, 1.3)	0.7 (0.0, 1.5)	0.0 (−2.4, 2.4)	−0.1 (−2.5, 2.3)
Birth weight in grams	No	17 154	3442 (637)	Ref	Ref	Ref	Ref
	Yes	632	3316 (698)	−118 (−168, −68)	−116 (−165, −67)	−109 (−210, −7)	−112 (−209, −15)
	Uncertain^d^	2451	3441 (648)	−2 (−29, 25)	9 (−18, 37)	−49 (−131, 34)	−23 (−103, 56)
Birth weight standardized *z*-score by sex, gestational age and parity	No	16 006	0.0 (1.0)	Ref	Ref	Ref	Ref
	Yes	583	−0.1 (1.0)	−0.1 (−0.2, −0.1)	−0.1 (−0.2, −0.1)	−0.1 (−0.3, 0.0)	−0.1 (−0.3, 0.0)
	Uncertain	2299	0.0 (1.0)	0.0 (−0.1, 0.0)	0.0 (−0.1, 0.0)	−0.1 (−0.2, 0.1)	−0.1 (−0.2, 0.1)

^a^The overall analyses was conducted using random-effects linear regression, which compares the means among all ART singletons with VTS to all ART singletons without VTS.

^b^The sibship analyses was conducted using fixed-effects linear regression, which compares the means ART singletons with VTS to their ART siblings without VTS.

^c^Adjusted for maternal age, marital status, parity, year of birth and chronic diseases before pregnancy (asthma, hypertension, heart disease, kidney disease, rheumatoid arthritis, epilepsy, thyroid disease and diabetes).

^d^When information from the early ultrasound was missing, the status of VTS was defined as ‘uncertain’.

The proportion of children born SGA was 9% (slightly less than 10% because the distributions were based on all deliveries in Norway within the study period). The sibling analysis of preterm birth included 587 ART singletons from 287 mothers who had at least one sibling discordant for preterm birth, while the sibling analysis of SGA included 674 ART singletons from 331 mothers with least one sibling discordant for SGA. Overall, ART singletons with VTS had an increased risk of being born SGA (Table III). The magnitude of this association was greater within sibships (Table III). There was weaker evidence for an association between VTS and risk of preterm birth (Table III).

**Table III:** The risk of preterm birth and SGA among ART singleton offspring with and without VTS, for all ART singletons born in Norway between July 1984 and December 2013.

Outcome	VTS	Overall analyses^a^			Sibship analyses^b^		
		*N* (% cases)	Unadjusted OR (95% CI)	Adjusted^c^ OR (95% CI)	*N* (% cases)	Unadjusted OR (95% CI)	Adjusted^c^ OR (95% CI)
Preterm birth (<37 weeks)	No	16 038 (9)	Ref	Ref	521 (50)	Ref	Ref
	Yes	583 (11)	1.29 (0.92, 1.80)	1.21 (0.87, 1.70)	20 (55)	1.21 (0.50, 2.92)	1.21 (0.49, 3.00)
	Uncertain^d^	2305 (9)	0.97 (0.80, 1.17)	0.91 (0.75, 1.10)	46 (46)	0.79 (0.37, 1.68)	0.83 (0.38, 1.79)
SGA (<10%)	No	16 006 (9)	Ref	Ref	584 (47)	Ref	Ref
	Yes	583 (12)	1.52 (1.10, 2.09)	1.48 (1.07, 2.03)	26 (69)	2.57 (1.06, 6.23)	2.79 (1.12, 6.91)
	Uncertain^d^	2299 (10)	1.18 (0.99, 1.42)	1.15 (0.96, 1.38)	64 (58)	1.76 (0.91, 3.41)	1.97 (0.99, 3.93)

OR, odds ratio; SGA, small for gestational age.

^a^The overall analyses was conducted using random-effects logistic regression, which compares the risk of the outcomes among all ART singletons with VTS to all ART singletons without VTS.

^b^The sibship analyses was conducted using fixed-effects logistic regression, which compares the proportion with VTS between ART singletons discordant for the outcome of interest.

^c^Adjusted for maternal age, marital status, parity, year of birth and chronic diseases before pregnancy (asthma, hypertension, heart disease, kidney disease, rheumatoid arthritis, epilepsy, thyroid disease and diabetes).

^d^When information from the early ultrasound was missing, the status of VTS was defined as ‘uncertain’.

In a sensitivity analysis that controlled for maternal smoking during pregnancy, the estimates remained largely unchanged (results not presented). The sensitivity analyses based on gestational age estimated using the date of birth minus date of ART procedure plus 14 days also produced similar results (results not presented). We also explored multivariable adjustment for ART treatment type (results not presented), and this did not change the results.

An alternative definition of VTS was based on the number foetuses with heart beats detected on early ultrasound. Although there was a higher proportion with missing information (27%), results were similar. The mean difference in birth weight between ART singletons with and without VTS was −93 g (−165, −20) and −129 g (−272, 14) when compared between and within sibships, respectively.

In general, ART pregnancies that started as a multiple pregnancy but lost one foetus were more like ART singleton pregnancies than ART multiple pregnancies. We compared the three groups of ART singletons (no vanishing twin, vanishing twin and uncertain status) to ART multiples. All three ART singletons groups had substantially higher gestational age and birth weight than ART multiples (Supplementary Table SI), and lower risk of preterm and SGA (Supplementary Table SII).

## Discussion

ART singletons with VTS had lower birth weight and greater risk of being born SGA than other ART singletons. These findings were robust within sibships, indicating that the associations are unlikely to be explained by unmeasured maternal background characteristics. Nevertheless, with regard to the pregnancy outcomes we evaluated, vanishing twin pregnancies seem to be more similar to ART singleton pregnancies than ART multiples.

### Strengths and limitations

The main strength of this study was the population-based design using a national registry, which provided a large study sample and the opportunity to compare siblings. The birth registry allowed us to include all ART deliveries in Norway, with the first ART delivery registered in 1984. Since the birth registry is the source of all national statistics for pregnancy outcomes in Norway, the distribution of pregnancy outcomes among ART singletons presented in this paper reflects the national average among all ART singletons.

Our study has some limitations. The birth registry does not contain information on pregnancy losses among naturally conceived pregnancies before 16 gestational weeks. Another limitation is a shortage of information on maternal lifestyle characteristics. By examining the associations within sibships, however, we were able to account for unmeasured lifestyle characteristics that remain stable across deliveries. The use of national registries for research purposes also relies on accurate registration of information. Validation studies from the birth registry indicate that gestational age and birth weight have high validity (Moth *et al.*, 2016). The number of ART singletons with VTS as identified through the birth registry was lower than estimated in some other study populations (Gjerris *et al.*, 2012). This may reflect a smaller number of embryos per transfer with IVF treatments in Norway.

There could also be period or cohort effects influencing the associations of interest. Our adjustment for maternal age and year of birth should account for such influences. We did not have sufficient numbers to explore this further in stratified analyses. Finally, we did not have information available to examine whether the associations between VTS among ART singletons and pregnancy outcomes could be influenced by fresh versus frozen embryo transfer. The proportion of VTS might be larger among offspring from fresh embryo transfer (Gu *et al.*, 2016). Furthermore, the risk of adverse pregnancy outcomes might be greater with fresh embryo transfers (Maheshwari *et al.*, 2016; Vidal *et al.*, 2017). It is therefore possible that the magnitude of the associations in our study are overestimated. Future studies should explore this possibility in more detail.

### Comparison with previous studies

Previous studies have compared pregnancy outcomes among ART singletons with VTS to either ART singletons without VTS or ART multiples (Pinborg *et al.*, 2005, 2007; Shebl *et al.*, 2008; Luke *et al.*, 2009; Almog *et al.*, 2010; Sazonova *et al.*, 2011; Davies *et al.*, 2016; Petrini *et al.*, 2016; Zhou *et al.*, 2016). The studies of ART singletons found reductions in birth weight with a vanishing twin ranging from 89 to 373 g (Pinborg *et al.*, 2005; Shebl *et al.*, 2008; Almog *et al.*, 2010; Sazonova *et al.*, 2011; Petrini *et al.*, 2016). Two studies reported an increased risk of SGA similar to our findings, with ORs of 1.50 and 2 (Pinborg *et al.*, 2007; Shebl *et al.*, 2008).

Some previous studies reported an association between VTS and gestational age/preterm birth, with differences in gestational age ranging from 4.2 days to 21.7 days (Pinborg *et al.*, 2005; Almog *et al.*, 2010), and the risk of preterm birth ranging from 1.5 to 3.9 (Pinborg *et al.*, 2005; Shebl *et al.*, 2008; Luke *et al.*, 2009; Almog *et al.*, 2010; Zhou *et al.*, 2016). Those differences were much smaller in our study, and did not reach statistical significance. We found that pregnancy outcomes among ART singletons with VTS were more similar to ART singletons without VTS than to ART multiples. This is consistent with most previous studies (Pinborg *et al.*, 2005; Zhou *et al.*, 2016), although not all (Almog *et al.*, 2010).

None of these previous studies provided a sibling comparison. By using a sibling comparison approach, we were able to show that the previously reported associations of VTS with birth weight and SGA are unlikely to be explained by maternal unmeasured background characteristics that remain stable between deliveries (Susser *et al.*, 2010; Frisell *et al.*, 2012). In contrast, the modest inverse association between VTS and gestational age, which was attenuated in the sibling analysis, seemed to be explained largely by such unmeasured confounding. Potential explanations include maternal socio-economic position, which was appropriately accounted for in the sibling analysis, and its association with various lifestyle characteristics that could influence the risk of an earlier delivery. Furthermore, since the method used to compare binary outcomes within sibships conditions the analyses on sibships with at least two siblings discordant for the outcome, the estimates reflect associations within a selected group. This needs to be considered when interpreting the associations. Even so, the findings from the sibship analyses do control for unmeasured factors and support our main conclusions.

### Potential mechanisms and future directions

It is well known that pregnancies resulting in a multiple birth have increased risk of all adverse pregnancy outcomes, and the risk profile of a pregnancy with multiples is closely related to placentation and number of chorions (Oepkes and Sueters, 2017). Residual problems of placentation may contribute to the reduced birth weight and increased SGA among ART singletons with VTS. There is some evidence linking VTS to pregnancy complications through absorption of necrotic fetoplacental tissue, which may result in increased cytokine and prostaglandin release and initiate an inflammatory process (Mansour *et al.*, 2010). Cytokines released from the vanishing twin may influence placental function, with subsequent consequences for the growth of the surviving foetus (Zhou *et al.*, 2016). Even so, the gestational age and birth weight among ART singletons with VTS were more similar to other ART singletons than to ART multiples.

Information on vanishing twins in naturally conceived pregnancies is not available in the Norwegian birth registry, since early ultrasound examinations are not part of routine antenatal health care. The associations we observed between VTS and pregnancy outcomes might be applicable to non-ART pregnancies with VTS, as suggested by the existing literature (Evron *et al.*, 2015).

The smaller birth size of ART singletons with VTS in our study is similar to the reduction observed with maternal smoking during pregnancy (Horta *et al.*, 1997; Blatt *et al.*, 2015). The reduced birth weight of babies with VTS might therefore be an indicator of an intrauterine environment with long-term health impact. Being born with low birth weight or SGA is associated with increased risk of chronic diseases such as asthma/allergies, neurodevelopmental disorders and metabolic disturbances linked to later cardiovascular disease (Murray *et al.*, 2015; den Dekker *et al.*, 2016; Mericq *et al.*, 2017). Our findings therefore support the idea that children born with VTS could be at increased risk of a broad range of chronic diseases. This could be explored further in large samples with follow-up information.

## Conclusion

In our large registry-based study, VTS among ART singletons was associated with lower birth weight and increased risk of SGA. These associations were not explained by unmeasured maternal background characteristics that remain stable between deliveries. The reduction in birth weight and increased SGA in ART singletons with VTS may be an indicator of an intrauterine environment that could have long-term health impact. VTS could potentially be a risk factor for all pregnancies, including those naturally conceived.

## Supplementary data

Supplementary data are available at *Human Reproduction* online.

## Supplementary Material

Supplementary Data

Supplementary Data
