# Supplementary material for: Vanishing twin syndrome among ART singletons and pregnancy outcomes
Source: Hum Reprod. 2017 Aug 31;32(11):2298–304. doi: 10.1093/humrep/dex277 (PMC5850786; doi:10.1093/humrep/dex277)
Supplement: Supplementary Data [file dex277suppl_table1.pdf]

Human Reproduction, 2017
doi:10.1093/humrep/dex277

human reproduction

SUPPLEMENTARY DATA

Supplementary Table SI Birth weight and gestational age among ART singletons with and without VTS compared to ART multiples, among all ART deliveries in Norway between July 1984 and December 2013.

Outcome	Exposure group	*N*	Mean (SD)	Overall analyses[a]		Sibship analyses[b]	
				Unadjusted	Adjusted[c]	Unadjusted	Adjusted[c]
				Mean difference (95% CI)	Mean difference (95% CI)	Mean difference (95% CI)	Mean difference (95% CI)
Gestational age in days	ART multiples	8279	252.3 (23.7)	Ref	Ref	Ref	Ref
	ART singletons without VTS	16 038	276.6 (16.6)	24.0 (23.4, 24.5)	23.8 (23.3, 24.4)	24.6 (23.5, 25.7)	24.29 (23.2, 25.4)
	ART singleton with VTS	583	275.0 (18.5)	22.9 (21.5, 24.3)	22.8 (21.4, 24.2)	24.6 (22.2, 26.9)	24.29 (22.0, 26.6)
	ART singleton with uncertain vanishing twin status[d]	2305	277.2 (16.7)	24.3 (23.5, 25.2)	24.4 (23.5, 25.2)	24.0 (22.1, 25.8)	23.97 (22.1, 25.8)
Birth weight in grams	ART multiples	8916	2462 (659)	Ref	Ref	Ref	Ref
	ART singletons without VTS	17 154	3443 (637)	954 (936, 973)	963 (945, 982)	890 (851, 929)	992 (952, 1032)
	ART singleton with VTS	632	3316 (698)	845 (796, 894)	854 (805, 902)	789 (704, 874)	891 (808, 974)
	ART singleton with uncertain vanishing twin status[d]	2451	3441 (648)	951 (922, 981)	973 (943, 1002)	864 (797, 930)	1000 (934, 1067)
Birth weight standardized *z*-score by gender, gestational age and parity	ART multiples	8188	−0.6 (0.8)	Ref	Ref	Ref	Ref
	ART singletons without VTS	16 006	0.0 (1.0)	0.7 (0.7, 0.7)	0.7 (0.7, 0.7)	0.7 (0.7, 0.8)	0.8 (0.7, 0.8)
	ART singleton with VTS	583	−0.1 (1.0)	0.6 (0.5, 0.6)	0.6 (0.5, 0.6)	0.5 (0.4, 0.7)	0.6 (0.4, 0.7)
	ART singleton with uncertain vanishing twin status[d]	2299	0.0 (1.0)	0.7 (0.6, 0.7)	0.7 (0.6, 0.7)	0.7 (0.6, 0.9)	0.8 (0.7, 0.9)

VTS, vanishing twin syndrome.
[a]The overall analyses was conducted using random-effects linear regression, comparing the means among the three groups of ART singletons to all ART multiples.
[b]The sibship analyses was conducted using fixed-effects linear regression, comparing the means among the three groups of ART singletons to their siblings who were ART multiples.
[c]Adjusted for maternal age, marital status, parity, year of birth and chronic diseases before pregnancy (asthma, hypertension, heart disease, kidney disease, rheumatoid arthritis, epilepsy, thyroid disease and diabetes).
[d]When information from the early ultrasound was missing, the status of VTS was defined as ‘uncertain’.
