# Supplementary material for: Vanishing twin syndrome among ART singletons and pregnancy outcomes
Source: Hum Reprod. 2017 Aug 31;32(11):2298–304. doi: 10.1093/humrep/dex277 (PMC5850786; doi:10.1093/humrep/dex277)
Supplement: Supplementary Data [file dex277suppl_table2.pdf]

Supplementary Table SII **The risk of preterm birth and small for gestational age (SGA) among ART singletons with and without VTS compared to ART multiples, among all ART deliveries in Norway between July 1984 and December 2013.**

Outcome	Exposure group	Overall analyses[a]			Sibship analyses[b]		
		N (% cases)	Unadjusted OR (95% CI)	Adjusted OR[c] (95% CI)	*N* (% cases)	Unadjusted OR (95% CI)	Adjusted OR[c] (95% CI)
Preterm birth (<37 weeks)	ART multiples	8279 (51)	Ref	Ref	572 (88)	Ref	Ref
	ART singletons without VTS	16 038 (9)	0.09 (0.09, 0.10)	0.10 (0.09, 0.11)	758 (37)	0.04 (0.03, 0.08)	0.04 (0.02, 0.06)
	ART singleton with VTS	583 (11)	0.12 (0.09, 0.15)	0.12 (0.09, 0.15)	31 (36)	0.05 (0.02, 0.12)	0.04 (0.02, 0.10)
	ART singleton with uncertain vanishing twin status[d]	2305 (9)	0.09 (0.08, 0.11)	0.09 (0.08, 0.11)	63 (35)	0.04 (0.02, 0.08)	0.03 (0.01, 0.07)
SGA (<10%)	ART multiples	8188 (23)	Ref	Ref	2486 (50)	Ref	Ref
	ART singletons without VTS	16 006 (9)	0.26 (0.24, 0.29)	0.26 (0.24, 0.29)	803 (40)	0.32 (0.23, 0.43)	0.30 (0.21, 0.43)
	ART singleton with VTS	583 (12)	0.39 (0.29, 0.53)	0.39 (0.28, 0.52)	35 (63)	0.84 (0.39, 1.84)	0.82 (0.37, 1.82)
	ART singleton with uncertain vanishing twin status[d]	2299 (10)	0.31 (0.26, 0.37)	0.31 (0.26, 0.37)	98 (40)	0.35 (0.20, 0.61)	0.33 (0.19, 0.59)

OR, odds ratio; VTS, vanishing twin syndrome.

[a]The overall analyses was conducted using random-effects logistic regression, which compares the risk of the outcomes among the three groups of ART singletons to all ART multiples.

[b]The sibship analyses was conducted using fixed-effects logistic regression, which compares the proportion of VTS among ART siblings discordant for the outcome of interest.

[c]Adjusted for maternal age, marital status, parity, year of birth and chronic diseases before pregnancy (asthma, hypertension, heart disease, kidney disease, rheumatoid arthritis, epilepsy, thyroid disease and diabetes).

[d]When information from the early ultrasound was missing, the status of VTS was defined as ‘uncertain’.
